# Total corpora mobilization for penile reconstruction

**DOI:** 10.1590/S1677-5538.IBJU.2022.0177

**Published:** 2022-05-18

**Authors:** Ubirajara Barroso, Bruna Venturini, Eliakim Massuqueto, Filip Prado, Ana Castro, Herbert Santos

**Affiliations:** 1 Universidade Federal da Bahia - UFBA Divisão de Urologia Salvador BA Brasil Divisão de Urologia, Universidade Federal da Bahia - UFBA, Salvador, BA, Brasil; 2 Escola Bahiana de Medicina e Saúde Pública Salvador BA Brasil Escola Bahiana de Medicina e Saúde Pública, Salvador, BA, Brasil

## Abstract

**Purpose:**

Total corpora mobilization (TCM) is a novel technique that is used for penile reconstruction in cases of micropenis and penile amputation. Its principle is based on Kelly’s procedure for bladder exstrophy (1). In contrast to the Kelly procedure, TCM is performed entirely through the perineum with the patient in the lithotomy position.

**Materials and Methods:**

TCM was performed on three patients. The first was a boy who suffered trauma from a dog bite at an age of eight months. At 23 years old he underwent TCM. The second patient had genital self-amputation induced by psychiatric disorder. After treatment, at 27 years old, he desired surgery for penile reconstruction. The third patient had partial androgen insensitivity syndrome (PAIS) with a micropenis and at 23 years old had TCM procedure.

The patients were placed in the lithotomy position with a perineal incision in the midline. A subperiosteal incision was made and the corpora cavernosa were detached from the pubic arch and the ischial rami. The periosteum and the neurovascular bundles were preserved. Subsequently the corpora cavernosa was mobilized upward and the periosteum that was left attached to them was sutured to the pubis.

**Results:**

At twenty-four, nine, and six months, respectively, in the follow-up process, all patients expressed satisfaction with the final cosmetic appearance, penile length, and erectile function.

**Conclusion:**

TCM may prove to be an alternative for patients with a functional disturbance because of small penile length, though a higher number of cases and a more extended follow-up are needed to draw a more definitive conclusion.

## References

[1] Kelly JH: Vesical exstrophy: repair using radical mobilisation of soft tissues. Pediatr Surg Int. 1995; 10(5–6):298–304.

